# Supplementary material for: Embodying Multifunctional Mechano‐Intelligence in and Through Phononic Metastructures Harnessing Physical Reservoir Computing
Source: Adv Sci (Weinh). 2023 Oct 23;10(34):2305074. doi: 10.1002/advs.202305074 (PMC10700179; doi:10.1002/advs.202305074)
Supplement: Supplementary file 1 — Supporting Information [file ADVS-10-2305074-s001.pdf]

ADVANCED SCIENCE
Open Access

Supporting Information

for *Adv. Sci.*, DOI 10.1002/advs.202305074

Embodying Multifunctional Mechano-Intelligence in and Through Phononic Metastructures Harnessing Physical Reservoir Computing

*Yuning Zhang, Aditya Deshmukh and Kon-Well Wang**

Supporting Information

Embodying Multifunctional Mechano-Intelligence in and through Phononic Metastructures Harnessing Physical Reservoir Computing

*Yuning Zhang, Aditya Deshmukh, K. W. Wang**
Department of Mechanical Engineering
University of Michigan
Ann Arbor, MI 48109, USA

***Corresponding author:** K. W. Wang (Kon-Well Wang), email: kwwang@umich.edu

Table of content:

Supplementary text
Supplementary Figures S1-S8

Supplementary Text

S1 Fabrication and experimental setups

Phononic metastructure fabrication. Figure S1A illustrates the phononic metastructure (PM) prototype, which consists of a chain of 11 periodic diatomic modules, as highlighted in **Figure S1C** and **Figure S1D**. Each PM module is composed of two rigid masses, m_1 and m_2, fabricated using rigid cast acrylic sheets. The masses are coupled with a pair of bent spring steel beams within each module. Additionally, the modules are interconnected using another pair of bent beams. To differentiate between m_1 and m_2, different colors of acrylic sheets are employed, with amber acrylic sheets representing m_1 and clear acrylic sheets representing m_2. Both types of acrylic sheets have a side length of 2.54cm, but different thicknesses of 3.175mm (amber) and 1.984mm (clear), respectively. The acrylic sheets are fabricated by laser cutting large acrylic plates (McMaster-Carr item number: 85635K421 and 8560K181) into small square sheets with holes for screws and hanging string loops.

The bent beams connecting m_1 and m_2 within a module are referred to as Beam I, and they are made of spring steel sheets with dimensions of 6.35cm in length, 1.27cm in width, and 0.102mm in thickness. The bent beams that interconnect the modules are designated as Beam II, and they are also made of spring steel sheets with dimensions of 6.35cm in length, 1.27cm in width, and 0.127mm in thickness. These spring steel sheets are fabricated by cutting 1095 spring steel strips (McMaster-Carr item number: 9075K4 and 9075K9) into the required sizes and punching screw holes.

The PM chain is assembled module by module using the precisely cut acrylic and spring steel sheets. As depicted in **Figure S1D**, a single PM module is assembled from four square acrylic sheets (one pair of amber acrylic sheets and one pair of clear acrylic sheets) and two spring steel sheets. During the assembly process, the spring steel sheets are first bent and then secured between the two pairs of acrylic sheets using plastic screws and nuts (McMaster-Carr item number: 95868A260 and 94812A200). The resulting PM modules have an initial length of $L_0 = 2.9\text{cm}$ when no external force is applied, and the rigid masses m_1 and m_2 are measured as $m_1 = 4.4\text{g}$ and $m_2 = 3.2\text{g}$ (including the nuts and screws).

To minimize friction, we secure two lubricated guiding metallic rods (McMaster-Carr item number: 5615N11) onto the testing table using rigid resin supports fabricated with a Formlabs Form 3+ 3D printer, and the PM is suspended beneath the guiding rods using plastic string loops (Amazon, brand: esowemsn, item number: MM00951(2)). This arrangement allows the PM to smoothly move and vibrate along the x direction. We fix the left end of the PM by gluing its leftmost rigid mass to the left supporter, and the rightmost mass is glued to the linear motion actuator that is firmly attached to the testing table using a 3D-printed support.

Wave source exciter. In our experimental setup, as shown in **Figure S1B**, we utilize an electromagnet (EM) copper coil shaker (Amazon, brand: Gikfun, item number: EK1909) attached to the m_1 of the leftmost fixed module. Additionally, three disk permanent magnets (McMaster-Carr item number: 5862K145) are affixed to the neighboring m_2. To power the EM coil shaker, we employ a waveform generator (Agilent 33522A) to generate harmonic voltage signals. Consequently, the input wave source the input wave source is in form of harmonic magnetic forces applied to the m_2 of the leftmost module.

Linear motion actuator. For the linear motion actuation, we utilize a linear motion actuator (Amazon, brand: ECO LLC, item number: L11TGF1000NB250-T-1) that operates at a constant travel speed of $v = 14\text{mm/s}$ when powered by a 12V DC power supply. The actuator's maximum achievable elongation is 250mm. The actuator is controlled by an L298N motor driver (Amazon, brand: Aideepen, item number: 70100755) and a microcontroller (Amazon, brand: Arduino, item number: A000066). These controlling components are powered by a constant 12V DC supply. We have developed an actuator control algorithm, enabling the actuator to generate any desired displacement X_a. That is, when a digital input X_a is provided, the actuator will be activated for a specific time interval $\Delta t = \frac{X_a}{v}$, resulting in the desired displacement X_a.

Measurements. To measure the steady-state wave dynamics along the PM, we employ a scanning laser Doppler vibrometer (LDV) (Polytec PSV-500). The LDV system captures the out-of-plane (y direction) vibration amplitudes at the mid-points of selected beams, outputting the measurements as analog voltages. These reservoir readouts are then transmitted to a LabView-based program, which performs the linear summation to obtain the final reservoir output X_a. This output is subsequently sent to the actuator control platform to guide its displacement.

Note: It is important to note that, only as a proof-of-concept basic research testbed demonstration, we used the LDV system and the LabView program for readout measurements and linear summation. Eventually, as a self-contained integrated device, physically embeddable flexible vibration sensors, such as piezoelectric film sensors[1,2], can be used for the readout measurements, and a simple analogue additive circuit can be used for signal linear summation. These prospects offer promising avenues for future studies.

S2 Numerical model

Mathematical formulation of bent beams' force-deformation relationship. To numerically simulate the wave propagation characteristics of the PM, we begin by mathematically formulating the nonlinear force-deformation relationships of the two types of bent beam elements. Our discrete model, as depicted in **Figure S2A**, represents each

bent beam in the module as two rigid rods. These pairs of rods are hinged at the center using torsional springs with a stiffness of k_θ, while their other ends are hinged to the rigid masses through torsional springs with a stiffness of $\alpha \cdot k_\theta$. These torsional springs effectively capture the bending stiffness of the spring steel beams. We assume that the rods have an initial rotation angle of θ_0 when no external force is applied. Consequently, the length of the rigid rods can be expressed as $L_r = \frac{L_0}{2\cos\theta_0}$, where L_0 is the initial length of the PM module. When the module is subjected to an external load F (positive representing stretching forces), the module will subject to a length change of ΔL and a rotation angle reduction of $\Delta\theta$. We can establish the torque balance of the rods using the following equation:

$$\frac{F}{2} \cdot L_r \cdot \sin(\theta_0 - \Delta\theta) = (1+\alpha) \cdot k_\theta \cdot \Delta\theta. \tag{S1}$$

Furthermore, the geometry of the system must satisfy the relationship:

$$L_r = \frac{L_0}{2\cos\theta_0} = \frac{L_0 + \Delta L}{2\cos(\theta_0 - \Delta\theta)}. \tag{S2}$$

By substituting Eq. (S1) into Eq. (S2), we can derive the nonlinear relationship between the loading force F and the deformation level ϵ, defined as $\epsilon = \frac{\Delta L}{L_0}$. This relationship is expressed as:

$$F(\epsilon) = k_{nl} \cdot \frac{\theta_0 - \arccos[(1+\epsilon)\cdot\cos\theta_0]}{\sqrt{\frac{1}{\cos^2\theta_0} - (1+\epsilon)^2}}, \tag{S3}$$

where $k_{nl} = \frac{4(1+\alpha)k_\theta}{L_0}$ is a constant that relates to the bending stiffness of the spring steel beams, and θ_0 is a geometry-related constant. Hence, once the k_{nl} and θ_0 are determined, the nonlinear force-deformation relationship of the bent beam elements can be mathematically formulated.

To find the values of k_{nl} and θ_0 for the two types of beams, we solve an optimization problem aimed at minimizing the total sum of the squared errors between the experimentally measured force-deformation profiles and the analytically fitted profiles. The optimization problem can be formulated as follows:

$$k_{nl}^{opt}, \theta_0^{opt} = \underset{k_{nl}>0,\theta_0\in(0,\frac{\pi}{2})}{\operatorname{argmin}} \sum_{i=1}^{M} \left(F(\epsilon_i) - F_{exp}(\epsilon_i)\right)^2. \tag{S4}$$

In Eq. (S4), the $\left(F_{exp}(\epsilon_i), \epsilon_i\right), i = 1, 2, \dots, M$, represent experimental measurements of the force-deformation profiles obtained through quasi-static tensile testing of single modules. The tensile testing is conducted using an Instron testing machine, as shown in **Figure S2B**, with a constant loading speed of 0.5 mm/min. The problem described in Eq. (S4) is then solved using the constrained nonlinear optimization package in MATLAB. The comparison between the experimental measurements and the optimal fitted analytical

force-deformation profiles is presented in **Figure 2A** of the main text, and excellent agreements are observed for both types of bent beams. For Beam I, the parameters are determined as $k_1^{opt} = 9.11N$ and $\theta_1^{opt} = 0.795\text{rad}$, while for Beam II, $k_2^{opt} = 20.86N$ and $\theta_2^{opt} = 0.812\text{rad}$. With these parameters, the analytical expression in Eq. (S4) can be used for the numerical studying of the PM wave dynamics.

Governing equations of the PM. The mathematical formulation of the bent beam elements allows us to simplify the PM chain into a lumped mass-nonlinear-spring model, as depicted in **Figure S2C**. In this model, we consider the masses of the PM modules as concentrated at the two rigid masses, m_1 and m_2. The bent beams are characterized as nonlinear springs with restoring force-deformation relationships as described by Eq. (S4). At the left end, the first rigid mass is fixed, and the second mass is excited with a harmonic force $F_{in} = A_0 \sin(2\pi f t)$, where A_0 is the input force amplitude and f is the excitation frequency. At the right end, a static global displacement X_a is applied to the rightmost mass. For an N −module PM ($N = 11$ in our testbed case), the equations of motion of the ith module from the left can be expressed as:

$$m_1 \frac{d^2 x_1^i}{dt^2} - F_1\left(\frac{x_2^i - x_1^i}{L_0}\right) + F_2\left(\frac{x_1^i - x_2^{i-1}}{L_0}\right) + \xi_1 \frac{dx_1^i}{dt} = 0, \tag{S5}$$

$$m_1 \frac{d^2 x_2^i}{dt^2} + F_1\left(\frac{x_2^i - x_1^i}{L_0}\right) - F_2\left(\frac{x_1^{i+1} - x_2^i}{L_0}\right) + \xi_2 \frac{dx_2^i}{dt} = 0. \tag{S6}$$

Here, x_1^i and x_2^i are the displacements of m_1 and m_2 in the ith module, respectively. $F_1(\epsilon) = F(\epsilon\,; k_1^{opt}, \theta_1^{opt})$ and $F_2(\epsilon) = F(\epsilon\,; k_2^{opt}, \theta_2^{opt})$ are the restoring forces provided by the two types of bent beams, i.e., Beam I and Beam II, based on Eq. (S3). ξ_1 and ξ_2 are the damping constants assigned to m_1 and m_2, accounting for the damping effects in the system and ensuring that the PM reaches a steady-state response. Eq. (S5) and Eq. (S6) is applicable to $i = 2, 3, \ldots, N$. Considering the left boundary and input excitation, the equations of motion for the first module are:

$$x_1^i = 0, \quad i = 1 \tag{S7}$$

$$m_1 \frac{d^2 x_2^i}{dt^2} + F_1\left(\frac{x_2^i - x_1^i}{L_0}\right) - F_2\left(\frac{x_1^{i+1} - x_2^i}{L_0}\right) + \xi_2 \frac{dx_2^i}{dt} = A_0 \sin(2\pi f t), \qquad i = 1 \tag{S8}$$

Considering the right boundary, Eq. (S6) for $i = N$ is changed to:

$$m_1 \frac{d^2 x_2^i}{dt^2} + F_1\left(\frac{x_2^i - x_1^i}{L_0}\right) - F_2\left(\frac{X_a - x_2^i}{L_0}\right) + \xi_2 \frac{dx_2^i}{dt} = 0, \qquad i = N. \tag{S9}$$

Then, with proper initial conditions, we numerically solve for Eq. (S5)-(S9) using MATLAB ODE45 package, which employs the Runge-Kutta method. Here, we set the initial positions of the masses to their equilibrium positions. Specifically, under a static

global deformation X_a, if the two types of bent beams are deformed by ΔL_1 and ΔL_2, respectively, the initial positions for m_1 and m_2 in the ith module can be determined as:

$$x_1^i\big|_{t=0} = (i-1)\cdot(\Delta L_1 + \Delta L_2), \tag{S10}$$

$$x_2^i\big|_{t=0} = i\cdot\Delta L_1 + (i-1)\cdot\Delta L_2. \tag{S11}$$

Here, ΔL_1 and ΔL_2 satisfy the following length and force balance relationships.

$$N\cdot(\Delta L_1 + \Delta L_2) = X_a, \tag{S12}$$

$$F_1\left(\frac{\Delta L_1}{L_0}\right) = F_2\left(\frac{\Delta L_2}{L_0}\right). \tag{S13}$$

Eq. (S12) and Eq. (S13) can be solved via the nonlinear equation solver in MATLAB, and the initial positions for the masses can then be determined. The initial velocities are set to be zero for all the masses in the PM.

Numerical simulation settings. For an input excitation with time period $T = 1/f$, we simulate the wave dynamics for a total time of 400 periods. To ensure accuracy and stability in the simulation, we use a constant time step size of $\frac{1}{300}T$. We set the damping constants to be $\xi_1 = \xi_2 = 3.5$, which allows the system to reach a steady-state after the initial 300 periods. To analyze the wave transmission characteristics of the incoming waves, we collect the velocity data during the last 100 periods of the simulation, which represents the steady-state behavior, and obtain their velocity amplitudes via Fast Fourier Transformation technique.

Transmission ratio definition. To quantify the wave transmission, we define the transmission ratio (TR) as the ratio of the average velocity amplitudes between the output modules and the input modules. In **Figure S2D**, the input modules are depicted as the two leftmost modules, while the output modules are represented by the two rightmost modules. During the simulation, we calculate the velocity amplitudes for the 8 masses within the input and output modules, which are used to determine the TR. In the experimental setup, we measure the velocity at the mid-points of the bent beams. Hence, when calculating the TR, we consider the velocity responses of the 8 pairs of bent beams within the input and output modules.

S3 Linear dispersion analysis

To gain further physical insight, we conduct linear dispersion analysis to theoretically derive the band structure of the PM under different global length configuration X_a. To this end, we firstly linearize the simplified mass-nonlinear-spring model under different global length configurations by linearizing each PM module around its equilibrium position. We then assume an unforced, undamped and infinite chain of such linearized PM module for to obtain the linear dispersion relations (see **Figure S2E**). Specifically, the linearized free wave equations of the ith linearized module can be written as:

$$m_1\frac{d^2x_1^i}{dt^2}+\tilde{k}_1\cdot(x_1^i-x_2^i)+\tilde{k}_2\cdot(x_1^i-x_2^{i-1})=0, \tag{S14}$$

$$m_2\frac{d^2x_2^i}{dt^2}-\tilde{k}_1\cdot\left(x_1^i-x_2^i\right)+\tilde{k}_2\cdot\left(x_2^i-x_1^{i+1}\right)=0. \tag{S15}$$

Here, $\tilde{k}_1$ and $\tilde{k}_2$ are the linearized stiffnesses of the two types of nonlinear springs under the deformation of ΔL_1 and ΔL_2, respectively. ΔL_1 and ΔL_2 can be determined by Eq. (S12) and Eq. (S13) for any given X_a. $\tilde{k}_1$ and $\tilde{k}_2$ can be derived based on Eq. (S3) by taking the derivative of the restoring forces with respect to the deformation. That is,

$$\tilde{k}_1=\frac{1}{L_0}\frac{dF_1}{d\epsilon}\bigg|_{\epsilon=\frac{\Delta L_1}{L_0}}, \tag{S16}$$

$$\tilde{k}_2=\frac{1}{L_0}\frac{dF_2}{d\epsilon}\bigg|_{\epsilon=\frac{\Delta L_2}{L_0}}. \tag{S17}$$

Based on the Bloch theorem[3], we assume solutions of Eq. (S14) and Eq. (S15) in the form of a traveling waves. That is, $x_1^i=U_1e^{j(\omega t-ika)}$, $x_2^i=U_2e^{j(\omega t-ika)}$, where j is the imaginary unit, k is the wave number, $\omega=2\pi f$ is the angular frequency, and a is the unit cell length constant. Substituting the solutions into Eq. (S14) and Eq. (S15) yields the following eigenvalue problem.

$$\omega^2\begin{bmatrix}m_1 & \\ & m_2\end{bmatrix}\begin{bmatrix}U_1\\U_2\end{bmatrix}=\begin{bmatrix}\tilde{k}_1+\tilde{k}_2 & -\tilde{k}_1-\tilde{k}_2e^{jka}\\ -\tilde{k}_1-\tilde{k}_2e^{-jka} & \tilde{k}_1+\tilde{k}_2\end{bmatrix}\begin{bmatrix}U_1\\U_2\end{bmatrix}. \tag{S18}$$

Then, the dispersion relation $\omega=\omega(k)$ can be determined by solving Eq. (S18), and the band structure can be obtained by sweeping k from 0 to π/a. It can be further determined that the bandgaps are the frequency ranges where no solution exists. That is, $[f_1,f_2]$ and $[f_3,+\infty]$, where the bandgaps boundary frequencies f_1, f_2 and f_3 are determined by Eq. (S19) - (S21).

$$f_1^2=\frac{(\tilde{k}_1+\tilde{k}_2)(m_1+m_2)-\sqrt{(\tilde{k}_1+\tilde{k}_2)^2(m_1+m_2)^2-16m_1m_2\tilde{k}_1\tilde{k}_2}}{8\pi^2m_1m_2}, \tag{S19}$$

$$f_2^2=\frac{(\tilde{k}_1+\tilde{k}_2)(m_1+m_2)+\sqrt{(\tilde{k}_1+\tilde{k}_2)^2(m_1+m_2)^2-16m_1m_2\tilde{k}_1\tilde{k}_2}}{8\pi^2m_1m_2}, \tag{S20}$$

$$f_3^2=\frac{(\tilde{k}_1+\tilde{k}_2)(m_1+m_2)}{4\pi^2m_1m_2}. \tag{S21}$$

With Eq. (S16), (S17), (S19) – (S21), the theoretical bandgaps of the PM under different actuator displacement X_a can be obtained.

S4 PM reservoir training and testing

To train the PM reservoir, we begin by selecting different input excitations as the training inputs. The inputs are chosen based on the specific task requirements. For frequency-

selective wave control tasks, we use inputs with different excitation frequencies, while for amplitude-selective wave control tasks, we fix the input frequency and vary the input amplitudes. The PM is excited with these training inputs, and we measure the steady-state velocity amplitudes of selected modules, which serve as the training data. For generalization, we consider M training inputs with excitation features I_i, $i = 1, 2, \dots, M$ (excitation frequency or amplitude). The corresponding targeted actuator displacements are denoted as $T(I_i), i = 1, 2, \dots, M$, which are known if the targeted mapping is designed and the training inputs are selected. For each input, let the PM reservoir readouts to be $A_{i1}, A_{i2}, \dots, A_{iN}$, which is the steady-state velocity amplitudes for N selected bent beams in the PM (see Section S7 for details on readout selections). Then, the final reservoir output is $X_i = \sum_{j=1}^{N} w_j A_{ij} + b$, where the readout weights $w_1, w_2, \dots, w_N$ and the bias b are the trainable parameters to be derived for different tasks. During the training phase, we determine the readout weights and bias by minimizing the error between the targeted displacements and the PM reservoir output across all the training samples. Here, we evaluate the training error using the Mean Square Error (MSE), defined as:

$$\mathrm{MSE}_{\mathrm{training}} = \frac{1}{M} \sum_{i=1}^{M} (X_i - T(I_i))^2, \tag{S22}$$

where the sum is evaluated over all the training samples. We can write the readouts for all the training samples into following data matrix $\mathbf{\Phi}$:

$$\mathbf{\Phi} = \begin{bmatrix} A_{11} & \cdots & A_{1N} \\ \vdots & \ddots & \vdots \\ A_{M1} & \cdots & A_{MN} \end{bmatrix}, \tag{S23}$$

where each row contains the readouts collected form one training input samples. Then, the problem can be expressed as the following optimization problem:

$$\boldsymbol{w}^{opt}, b^{opt} = \underset{\boldsymbol{w}, b}{\operatorname{argmin}} \|\mathbf{\Phi} \cdot \boldsymbol{w} + \boldsymbol{b} - \boldsymbol{X_T}\|_2^2, \tag{S24}$$

where $\|\cdot\|_2$ represents the Euclidean norm of vectors, $\boldsymbol{w} = [w_1\, w_2 \dots w_N]^T$ represents the output weightings, $\boldsymbol{b} = [b\, b \dots b]^{\boldsymbol{T}}$ is the bias in vector form, and $\boldsymbol{X_T} = [T(I_1)\, T(I_2) \dots T(I_M)]^T$ is a vector containing the targeted displacements. This problem is an ordinary least square regression problem and has the closed-form solution as shown in Eq. (S25).

$$\left[w_1^{opt}\, w_2^{opt} \dots w_N^{opt}\, b^{opt}\right]^T = \tilde{\mathbf{\Phi}}^{\dagger} \cdot \boldsymbol{X_T}, \tag{S25}$$

where $\tilde{\mathbf{\Phi}} = [\mathbf{\Phi}\ \mathbf{1}]$ is the augmented data matrix with an added column of ones, and $[\cdot]^{\dagger}$ refers to the Moore-Penrose pseudo-inverse of a matrix.

After obtaining the optimal readout weightings of a task, we evaluate the performance of the PM reservoir on S unseen testing inputs, and the testing error can be obtained via MSE over all the testing samples. That is,

$$\mathrm{MSE_{test}} = \frac{1}{S}\sum_{i=1}^{S}(X_i - T(I_i))^2. \tag{S26}$$

S5 Self-adaptive wave transmission control tasks

Frequency-selective wave transmission control. The frequency-selective transmission control is achieved by designing frequency-dependent targeted input-output mappings, denoted as $X_T = T(f)$. In our study, we focus on an operating frequency range of 80Hz to 136Hz, where the PM initially exhibits a stopband between 80Hz and 114Hz and a passband between 114Hz and 136Hz. We investigate two tasks: a self-adaptive wave blocking task and a self-adaptive wave passing task.

For the wave blocking task, we have already presented the results in **Figure 3A** of the main text. The targeted displacements for this task are represented by the black dots in **Figure 3A**, which are all located within the initial bandgap to prevent wave propagation. The targeted input-output mappings for this task can be expressed using Eq. (S27). Specifically, for input frequencies within the initial bandgap, i.e., between 80Hz and 114Hz, the targeted displacements are set to zero since the wave is already prohibited. For input frequencies within the initial passband, the targets are set to be located along a straight line within the bandgap.

$$X_T(f) = \begin{cases} 0, & f \in (80\text{Hz}, 114\text{Hz}) \\ 0.193f - 14.57, & f \in (114\text{Hz}, 136\text{Hz}) \end{cases} \tag{S27}$$

The targeted input-output mappings for the wave passing task are shown in **Figure S3**. In this task, the PM is trained to allow the passage of all input waves, irrespective of their frequencies. The targeted mappings are designed to be within the passband of the PM, enabling the input waves to propagate through. The specific mapping for this task can be described using Eq. (S28). For input frequencies within the initial passband, the targeted displacements are set to zero, and input frequencies within the initial bandgap, the targets are set to be located along a straight line within the passband.

$$X_T(f) = \begin{cases} 0.094f + 1.99, & f \in (80\text{Hz}, 114\text{Hz}) \\ 0, & f \in (114\text{Hz}, 136\text{Hz}) \end{cases} \tag{S28}$$

The results in **Figure S3** demonstrate the successful implementation of the wave passing task, as indicated by the final positions of all the training and testing samples falling within the passband. The TR plot also reveals that for input frequencies originally within the bandgap, the TR of the incoming wave significantly increases due to the self-tuning of the PM to allow wave passage.

Amplitude-selective wave transmission control. Amplitude-selective wave transmission control is achieved by designing amplitude-dependent targeted input-output mappings, denoted as $X_T = T(A_0)$. In **Figure 3C** of the main text, we demonstrate the realization of

an unconventional wave filter that selectively allows the transmission of moderate-amplitude input waves. For this task, we set the targets for input amplitudes between 3.33V and 6.67V to a constant value of 10.2 cm within the bandgap region. Input amplitudes below 3.33V and above 6.67V are targeted to be zero, effectively blocking the transmission of low and high-amplitude waves.

In addition to this task, we implement two more amplitude-selective wave propagation control task as illustrated in **Figure S4A** and **Figure S4B**. In **Figure S4A**, input waves with amplitudes lower than 5V are blocked, while input waves with amplitudes ranging from 5V to 10V are allowed to pass through the PM. On the other hand, the task in **Figure S4B** demonstrates an inverse wave control strategy, where high-amplitude waves are blocked, and only low-amplitude waves are transmitted. These examples showcase the efficiency and versatility of the proposed PM reservoir framework in realizing multiple tasks using the same PM structure.

S6 Wave-based logic gates

Single-input logic gates. The implementation of single-input logic gates is achieved through the amplitude-selective wave control tasks I and II, as described in Section S5. In these tasks, we define binary inputs based on the input wave amplitudes, where input amplitudes smaller than 5V represent the '0' bit, and input amplitudes higher than 5V represent the '1' bit. The logic gate outputs are determined by the TRs, where a low TR corresponds to the '0' state (wave blocking and gate 'OFF'), and a high TR corresponds to the '1' state (wave propagation and gate 'ON').

Based on this definition, the amplitude selection task I shown in **Figure S4A** can function as a BUFFER gate. It blocks low-amplitude waves (input '0') and allows high-amplitude waves (input '1') to pass through, thereby representing gate 'OFF' for '0' input and gate 'ON' for '1' input. Similarly, the amplitude selection task II in **Figure S4B** serves as a NOT gate. It blocks high-amplitude input waves (input '1') and allows low-amplitude waves (input '0') to pass, representing gate 'OFF' for '1' input and gate 'ON' for '0' input.

Two-input logic gates. Two-input logic gates are implemented numerically using a doubled-PM reservoir, as illustrated in **Figure S5A**. Two identical PMs are arranged in parallel, where their left ends are fixed to the same position and their first modules on the left are independently excited with two incoming waves. The right ends of both PMs are connected to the same linear motion actuator, resulting in a two-input-one-output system. Similar to the single-input logic gates, we define logic inputs and outputs based on input wave amplitudes and TRs, respectively. In the simulation, we independently excite the two PMs with two harmonic forces applied to their first modules, using a fixed excitation frequency of 125Hz. The input amplitudes are selected between 1×10^{-4} N and 0.2 N.

The logic inputs A and B are defined based on the excitation amplitudes of the two input waves, where amplitudes below 0.1 N represent '0' bits and amplitudes above 0.1 N represent '1' bits. The gate outputs are determined by the average TRs of the two PMs.

We utilize 1000 different inputs for our study, with 250 inputs randomly generated for each combination of binary inputs (A, B). These inputs are indexed from 1 to 1000, with 1-250 representing an input of (0, 0), 251-500 representing (0, 1), 501-750 representing (1, 0), and 751-1000 representing (1, 1). These inputs are randomly split into 800 training samples and 200 testing samples. These inputs are randomly split into 800 training samples and 200 testing samples. By leveraging the wave dynamics of both PMs as a physical reservoir, we train the system to realize all six basic two-input logic gates: AND, OR, NAND, NOR, XOR, and XNOR. The truth table for these gates is shown in **Figure S5B**.

The training and testing results are presented in **Figure S5C** to **Figure S5H**. For each logic gate task, a targeted mapping is designed (represented by the black dots) to reconfigure the double-PM system and either block or pass the incoming wave, corresponding to the desired logic outputs. For example, for AND gates, the targeted displacements for inputs (0, 0), (0, 1), and (1, 0) are located within the bandgap to block the wave and result in an 'OFF' gate, while the targets are set within the passband for input (1, 1), resulting in an 'ON' gate.

S7 LASSO regression for readout selections

To further reduce reliance on electronics and digital components, we incorporate the Least Absolute Shrinkage and Selection Operator (LASSO) technique for readout selections. LASSO is a widely used regression analysis method in statistics and machine learning that performs variable selection and regularization, improving prediction accuracy and interpretability of regression models[4,5]. It selects a reduced set of variables to be used in the model.

In our case, with an 11-module PM chain, there can be up to 22 potential readouts from the 22 pairs of bent beams. LASSO helps identify the most informative readouts while eliminating redundant ones for wave control tasks. By applying LASSO, we can enhance the efficiency and effectiveness of the PM reservoir by selecting a smaller subset of readouts that are essential for achieving the desired control tasks. In our approach, we apply LASSO on the training dataset and solve the following constrained regression problem, as expressed in Eq. (S29).

$$\min_{\boldsymbol{w},\boldsymbol{b}} \|\boldsymbol{\Phi}\cdot\boldsymbol{w}+\boldsymbol{b}-\boldsymbol{X_T}\|_2^2, \quad \text{subject to} \quad \sum_{i=1}^{N}|w_i| \le c, \tag{S29}$$

where c is a given free parameter that determines the level of regularization. This problem can be reformulated in the Lagrangian form as shown in Eq. (S30):

$$\min_{\boldsymbol{w},\boldsymbol{b}} \|\boldsymbol{\Phi} \cdot \boldsymbol{w} + \boldsymbol{b} - \boldsymbol{X_T}\|_2^2 + \lambda \cdot \|\boldsymbol{w}\|_1 , \quad \text{(S30)}$$

where $\|\boldsymbol{w}\|_1 = \sum_{i=1}^{N} |w_i|$ represents the L_1 norm, which is the sum of the absolute values of its elements, and λ is a hyperparameter that needs to be specified. LASSO regression provides a sparse solution for $\boldsymbol{w}$, with many of the weightings being zero. This means that readouts with zero weightings do not contribute to the final reservoir outputs, allowing us to remove these readouts from consideration. Choosing the regularizartion hyperparameter λ is a fundamental part of LASSO. Typically, the sparsity of the solution depends on the value of λ, with larger values of λ leading to sparser solutions. In our case, we employ a two-step process to achieve a specific task with a reduced number of readouts. In the first step, we employ the built-in LASSO solver in MATLAB to solve Eq. (S30) for different values of λ and select the readouts with non-zero weightings for each choice of λ. In step two, these LASSO-selected readouts will serve as the physical reservoir for training the PM by solving Eq. (S24), and the optimal readout selection can be determined by comparing the reservoir testing performace under different choice of λ.

As an example, for the wave blocking task as shown in **Figure 3A** (main text), we firstly perform LASSO regression with different values of λ. **Figure S6A** displays the number of LASSO-selected readouts for different λ. As anticipated, as λ increases, the number of selected readouts gradually decreases to 1. Subsequently, in the second step, we train the PM reservoir for the wave blocking task using different numbers of LASSO-selected readouts, and evaluate the reservoir performace with testing dataset. **Figure S6B** shows the training and testing errors, measured in terms of MSE, for different numbers of LASSO-selected readouts. It is observed that the training error increases as the number of readouts decreases. However, the testing error fluctuates initially and reaches a minimum value when 15 readouts are selected, after which it starts to increase as the number of readouts decreases further. This suggests that it is possible to reduce the number of readouts without compromising the computational accuracy of the physical reservoir. Consequently, the optimal readout selection, considering all possible numbers of readouts, can be determined based on the testing performance of the PM reservoir across all testing samples. In the case of the wave blocking task, the 15-readout case is identified as the optimal choice based on the minimal testing MSE. To further assess the effectiveness of this technique, we compare the performance of the optimal LASSO-selected reservoirs (with 15 readouts) with that of random selections. We randomly select different number readouts from the total of 22 (200 ramdon selections for each fixed number of readouts). **Figure S6C** illustrates the testing performance based on the over 4000 random data selections compared to the optimal selection via LASSO. The black dots represent the various random selections of readouts, while the blue dash line represents the optimal testing MSE achieved via LASSO

regression with 15 readouts. It is observed that the optimal selection via LASSO achieves a better testing performance than almost all the random selections, although the LASSO solver was applied only to the training dataset in step one of the process. This demenstrates that LASSO provides the optimal choice of readouts for achieving the best reservoir performance. We also apply the readout selection technique using LASSO regression to other tasks and find that the optimal readout selection can vary for different tasks. For example, the wave-passing task achieves the best testing performance when all 22 readouts are used, while the wave-selective task in **Figure 3C** (main text) utilizes 16 readouts.

To gain a deeper understanding of how different numbers of readout selection influences the performance of the PM reservoir, we conduct numerical studies on a 30-module PM. In this case, the velocity amplitudes of the 60 masses in the PM are collected as available readouts. We use the wave-blocking task as an example. **Figure S7A** illustrates the number of LASSO-selected readouts for different values of λ, while **Figure S7B** shows the training and testing errors for the wave-blocking task with varying numbers of selected readouts. Consistent with the previous case, the training error increases as the number of readouts decreases. The testing error initially decreases to a minimum value and then increases as the number of readouts further decreases. It is also observed that when most of the readouts are selected, such as all 60 readouts (**Figure S7E**), the training error is significantly less than the testing error, indicating an overfitting phenomenon due to an excessive number of variables in the regression problem. The PM reservoir achieves the best testing performance when 18 readouts are selected, as both the training and testing samples accurately capture the desired targets (**Figure S7D**). These results demonstrate that LASSO regression can effectively perform variable selection and mitigate overfitting. Furthermore, we observe that even with as few as 5 selected readouts in this case (**Figure S7C**), an acceptable performance can still be achieved, where the final positions of all the training and testing samples lie within the bandgap, indicating successful wave blocking.

In real-world implementation and/or experimental investigations, we will have manufacturing defects, test errors, and inherent noise. Our experimental studies on the research testbeds have shown very good results, indicating the system is robust against such uncertainties and noise. However, to gain more understanding and account for these factors, we introduce random white noise into the readouts during the numerical studies of the 30-module PM case study for the wave blocking task. In our study, noisy data are generated by adding random values drawn from a fixed range of $[0, A_{noise}]$ to the reservoir readouts. The impact of noise on the reservoir performance is characterized in **Figure S8A**, where the readout selections are fixed as in **Figure S7D** (18 readouts), and the noise level is defined as the ratio between the maximum noise amplitude A_{noise} and the average mass velocity amplitude. The results reveal that noise in the system impairs the performance of the PM reservoir. We also perform readout selection via LASSO for a specific noise level

of $\epsilon_n = 0.05$. **Figure S8B** presents the training and testing errors for different numbers of LASSO-selected readouts. The optimal testing MSE is found to be 1.16 when 35 readouts are selected. In comparison, the case without noise shown in **Figure S7B** achieves an optimal test MSE of 0.76 with 18 readouts selected. The results indicate that the optimal testing performance decreases in the presence of noise, necessitating a higher number of readouts to achieve the desired reservoir performance. Therefore, by reducing the noise level in the system, we can improve the overall system performance with less readouts.

Supplementary References

[1] J.H. Han, K.H. Rew, I. Lee, *Smart Mater. Struct.* **1997**, *6*, 549.

[2] J.H. Song, Y.T. Kim, S. Cho, W.J. Song, S. Moon, C.G. Park, S. Park, J.M. Myoung, U. Jeong, *Adv. Mater.* **2017**, *29*,1702625.

[3] A.S. Phani, M.I. Hussein, *Dynamics of lattice materials*, John Wiley & Sons Ltd, Chichester, West Sussex, United Kingdom **2017**.

[4] R. Tibshirani, *J. R. Stat. Soc. Ser. B Methodol.* **1996**, *58*, 267.

[5] R. Muthukrishnan, R. Rohini. in *Proc. 2016 IEEE international conference on advances in computer applications (ICACA)*, **2016**, 18.

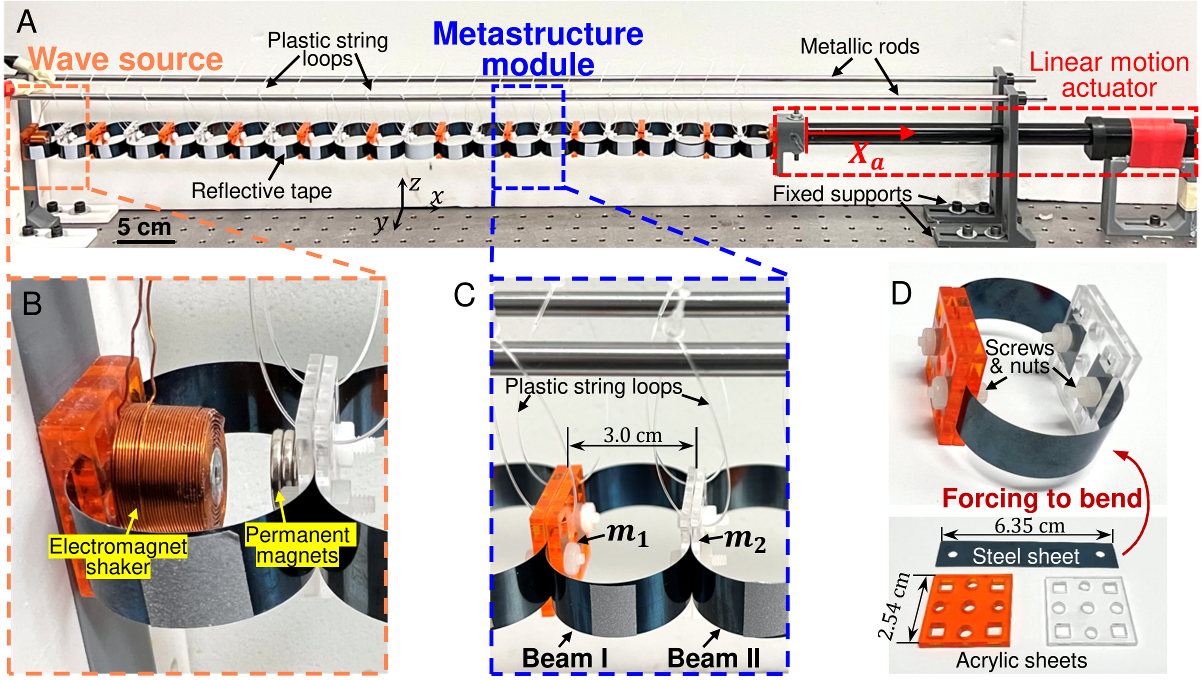

Figure S1. Phononic metastructure prototype. (A) An 11-module PM chain with an embedded linear motion actuator to alter its length configuration; (B) Wave source via an electromagnetic coil shaker; (C) A single PM module; (D) Assembly of the PM modules.

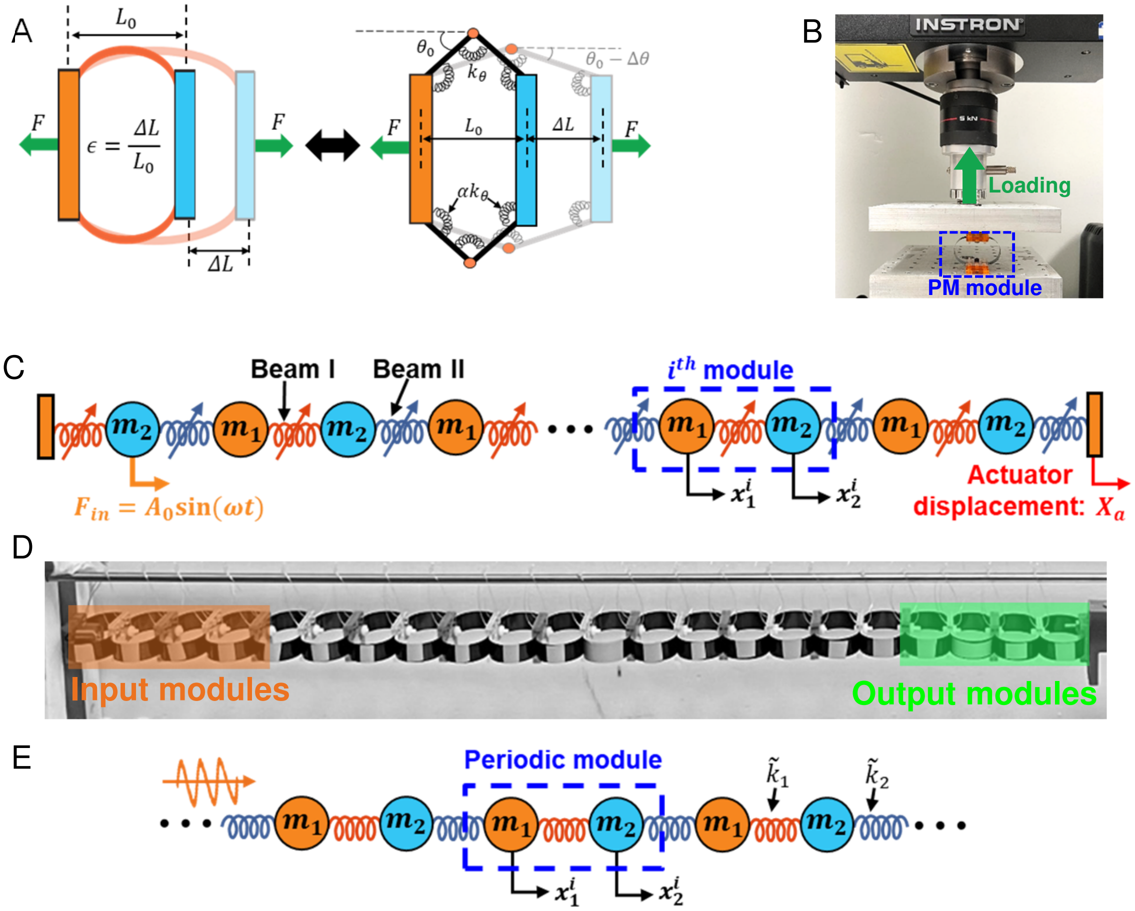

Figure S2. Analytical and numerical models. (A) Discrete model for a single module; (B) Tensile testing of a PM module using Instron Machine; (C) Lumped mass-nonlinear-spring model; (D) Definition for input and output modules; (E) Infinite chain of periodic modules for linear dispersion analysis.

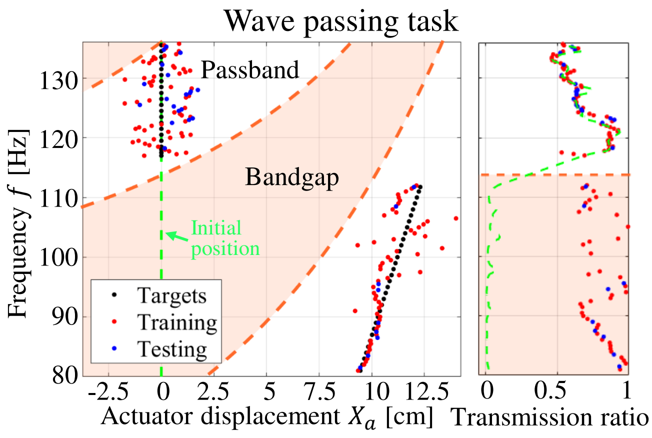

Figure S3. Self-adaptive wave passing task

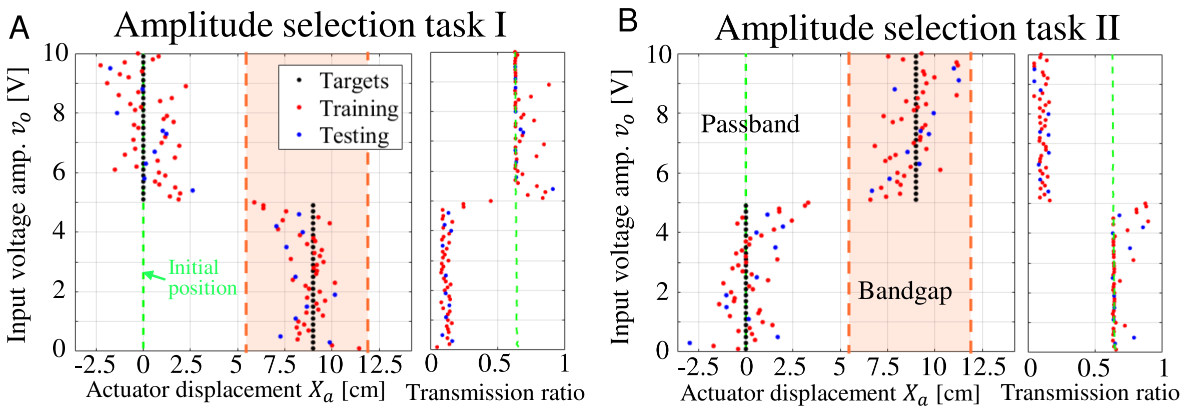

Figure S4. Self-adaptive amplitude-selective wave control. (A) PM blocks the low-amplitude waves and passes high-amplitude waves. (B) PM passes the low-amplitude waves and blocks high-amplitude waves.

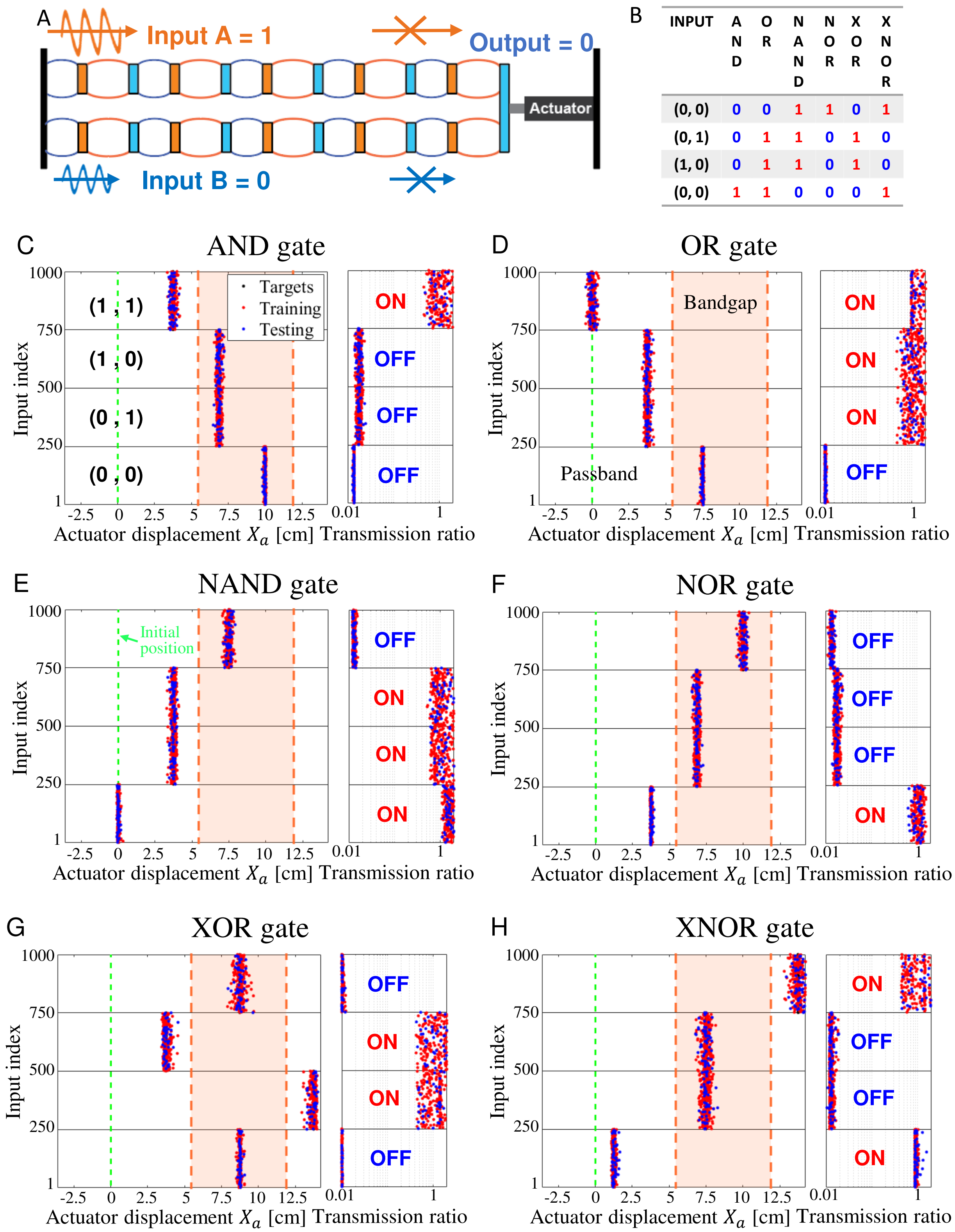

Figure S5. Two-input wave logic gates. (A) A conceptual double-PM system by assembling two identical PM in parallel; (B) Truth table for the six basic two-input logic gates; (C)-(H) Training and testing results for AND, OR, NAND, NOR, XOR, XNOR gates.

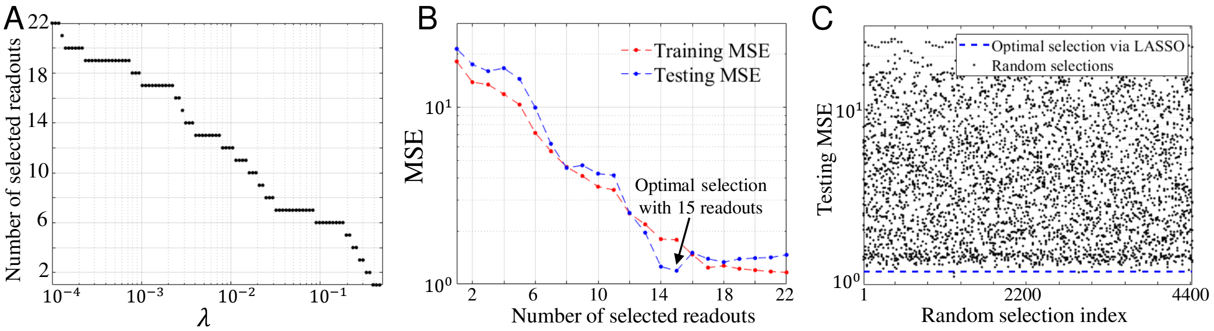

Figure S6. Readout selection in experiments via LASSO. (A) Number of LASSO-selected readouts for different values of λ; (B) Training and testing errors in MSE when training with different numbers of LASSO-selected readouts. (C) Testing MSEs for the wave blocking task under random selections of different numbers of readouts compared with optimal selection via LASSO. Black dots represent various random selections, and the blue dash line represents the optimal testing MSE achieved via LASSO regression with 15 readouts.

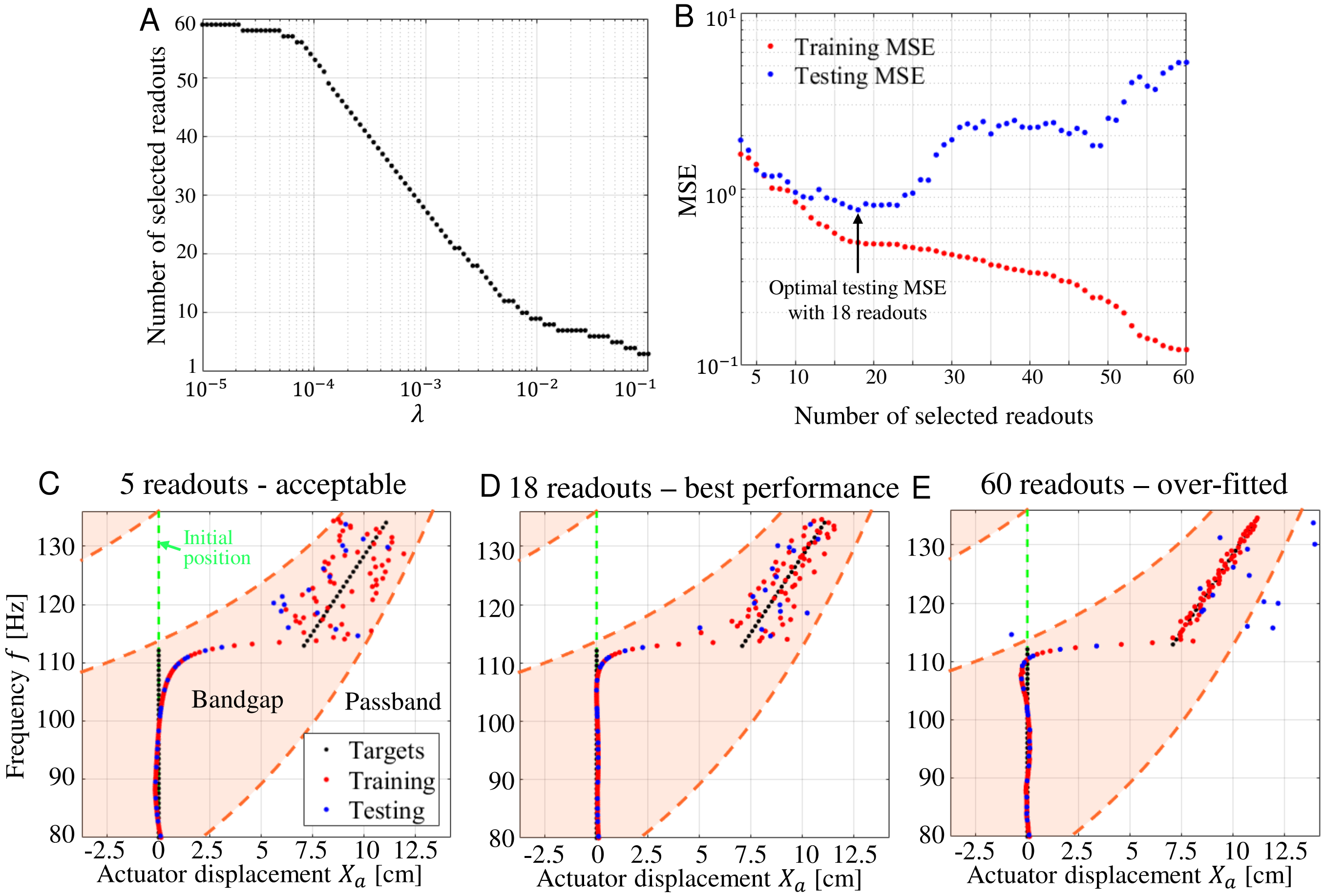

Figure S7. Numerical studies on readout selection via a 30-module PM. (A) Number of selected readouts for different values of λ; (B) Training and testing errors in MSE when training with different numbers of LASSO-selected readouts; (C)-(E) Training and testing results for wave blocking tasks when the number of readouts is 5 (C), 18 (D), and 60 (E).

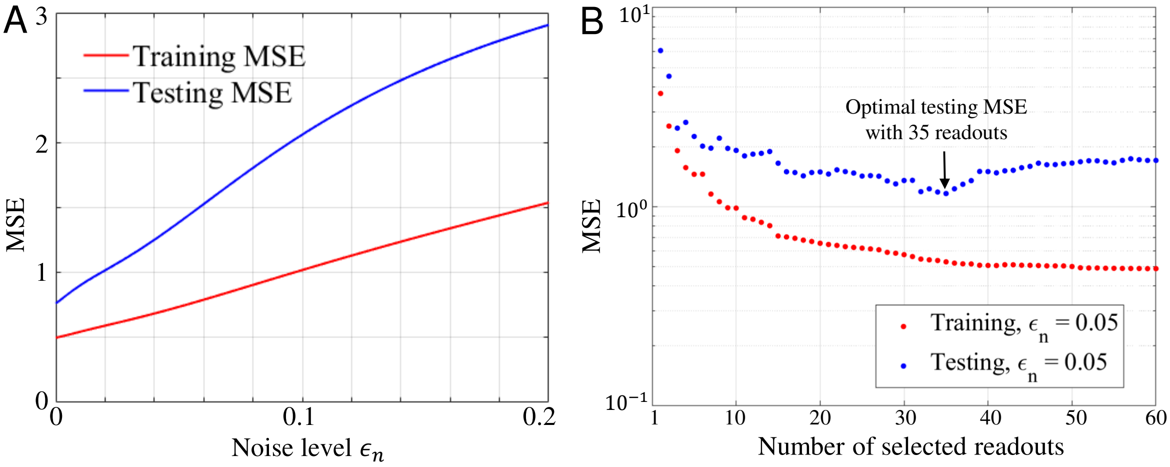

Figure S8. Effect of noise level on PM reservoir performance. (A) Training and testing MSEs of the wave blocking task when different levels of noise are added. (B) Training and testing MSEs with different numbers of LASSO-selected readouts for noisy data with a noise level of 0.05.
